# Precision Targets for Intercepting the Lethal Progression of Prostate Cancer: Potential Avenues for Personalized Therapy

**DOI:** 10.3390/cancers14040892

**Published:** 2022-02-11

**Authors:** Max Christenson, Chung-Seog Song, Ya-Guang Liu, Bandana Chatterjee

**Affiliations:** Department of Molecular Medicine, Long School of Medicine, University of Texas Health San Antonio, San Antonio, TX 78229, USA; christensonm@livemail.uthscsa.edu (M.C.); songc@uthscsa.edu (C.-S.S.); liuy9@uthscsa.edu (Y.-G.L.)

**Keywords:** high-risk prostate cancer, androgen receptor axis, castration resistance, neuroendocrine carcinoma, PARP inhibition, immunotherapy, cancer genome, precision targeting

## Abstract

**Simple Summary:**

Metastatic prostate cancer is incurable and lethal. Tumor growth is initially reduced by radiation, surgery, or hormone therapy and later, by pairing them with chemotherapy for advanced cancer. Recent innovations have helped to develop prescription drugs against certain prostate cancer types, showing gene alterations that prevent the repair of damaged DNA or activate the body’s anti-cancer natural immune defense. A panel of genes has been identified whose cancer genome alterations may predict whether non-metastatic prostate cancer would go on to metastasize. The activity of these genes may help to guide treatment decision for non-metastatic cancer with the choice for non-aggressive versus debilitating aggressive options. The probing of prostate cancer genome has uncovered hormonal abnormalities and genome changes specific to individual patients and studies are revealing how these changes can lead to treatment failure. The discovery of new druggable vulnerabilities of the cancer cells has presented opportunities to develop precision treatments of metastatic prostate cancer tailored to individual patients.

**Abstract:**

Organ-confined prostate cancer of low-grade histopathology is managed with radiation, surgery, active surveillance, or watchful waiting and exhibits a 5-year overall survival (OS) of 95%, while metastatic prostate cancer (PCa) is incurable, holding a 5-year OS of 30%. Treatment options for advanced PCa—metastatic and non-metastatic—include hormone therapy that inactivates androgen receptor (AR) signaling, chemotherapy and genome-targeted therapy entailing synthetic lethality of tumor cells exhibiting aberrant DNA damage response, and immune checkpoint inhibition (ICI), which suppresses tumors with genomic microsatellite instability and/or deficient mismatch repair. Cancer genome sequencing uncovered novel somatic and germline mutations, while mechanistic studies are revealing their pathological consequences. A microRNA has shown biomarker potential for stratifying patients who may benefit from angiogenesis inhibition prior to ICI. A 22-gene expression signature may select high-risk localized PCa, which would not additionally benefit from post-radiation hormone therapy. We present an up-to-date review of the molecular and therapeutic aspects of PCa, highlight genomic alterations leading to *AR* upregulation and discuss AR-degrading molecules as promising anti-AR therapeutics. New biomarkers and druggable targets are shaping innovative intervention strategies against high-risk localized and metastatic PCa, including AR-independent small cell-neuroendocrine carcinoma, while presenting individualized treatment opportunities through improved design and precision targeting.

## 1. Introduction

As the second most common male cancer worldwide, next only to lung cancer, prostate cancer affects roughly 1.3 million people and kills more than 360,000 people annually. In the U.S.A., the year 2020 saw nearly 192,000 new cases of prostate cancer (PCa) along with around 33,000 deaths [1]. Early stage PCa is treatable, with a 5-year survival rate of about 95%. Once PCa metastasizes, the 5-year survival rate falls to less than 30%. Conventional treatments for early stage PCa include watchful waiting (i.e., observation/no therapy), surgery and radiation, or active surveillance entailing routine monitoring of the blood level of prostate specific antigen (PSA), a circulatory biomarker for PCa cells, and repeat biopsy when deemed necessary. Cancer re-emergence occurs in about 30% of cases following radiation and/or surgery when hormone therapy, which reduces blood androgens to a castrate level, initially prevents androgen-dependent tumor growth. Hormone therapy is also considered for early stage cancer categorized as high risk for progression based on tumor grade and stage, and blood PSA. Hormone therapy and chemotherapy are the standard of care for PCa that has advanced locally or spread to distant tissues. No treatment course can offer permanent remission of advanced PCa. Technological advances are leading the way to find novel avenues for clinical interventions. In this review, we summarize progress in the molecular and genomic characterization of various stages of PCa—from high-risk localized and metastatic disease to neuroendocrine carcinoma, which is a highly aggressive form of advanced PCa—describe how insights gleaned from ongoing studies are revealing new vulnerabilities of cancer cells, and highlight several potential targets that are being pursued for future interventions tailored to individual patients.

In 1941, Charles Huggins discovered revolutionary aspects of PCa by demonstrating reduced cancer progression for the metastatic disease via androgen deprivation therapy (ADT) in the form of surgical castration. The Huggins discovery pioneered PCa management employing ADT and additional forms of hormone therapy, while also paving the way for a multitude of modern-day therapeutics [2]. Androgens play essential roles in the development of normal and malignant prostate, and the androgen dependence of PCa for cancer cell survival and proliferation has been amply documented. Androgens, synthesized predominantly in the testes, mediate biological effects via the androgen receptor (AR), which is a nuclear receptor and ligand-inducible transcription factor. Androgen-activated AR regulates normal prostate physiology and prostate cancer pathophysiology [3,4,5,6].

**Figure 1:**
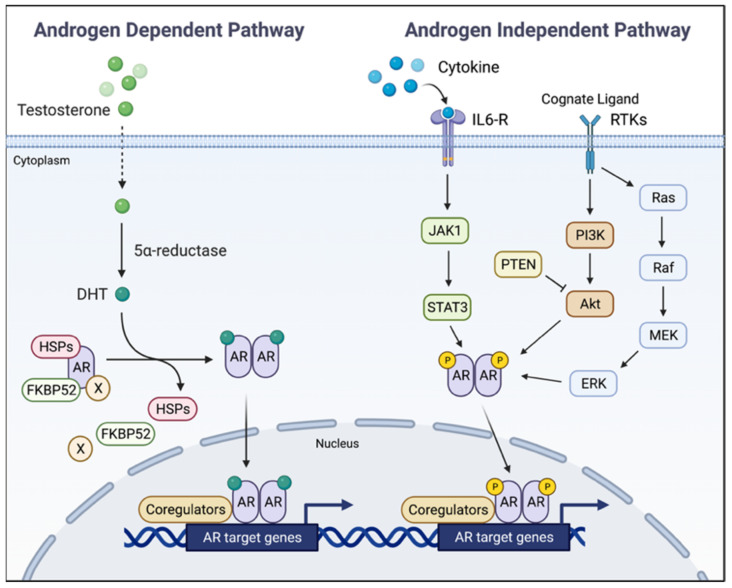
Simplified schema for AR-mediated target gene transcriptional response. DHT-bound AR dissociates from chaperone proteins (HSPs) and other interacting proteins, exposing AR’s nuclear localization signal and facilitating receptor’s nuclear import. Upon recruitment to androgen response elements of AR target genes, the hormone-bound AR interacts with various coregulators leading to target gene transcriptional response. An androgen-independent pathway may also drive AR activation and its cytoplasm to nucleus transfer subsequent to AR phosphorylation by various kinases. DHT: 5α-dihydrotestosterone; HSPs: heat shock proteins; FKBP52: forskolin-binding protein 52; IL6-R: interleukin 6 receptor; RTK: receptor tyrosine kinase.

Androgen precursors in the form of androstenedione and dehydroepiandrosterone are secreted into circulation following biosynthesis in the adrenal glands. Adrenal androgens are converted to testosterone intratumorally in the prostate via multiple enzymatic steps, and 5α-dihydrotestosterone (DHT) is the reduced metabolite of testosterone, generated by the catalytic action of 5α-reductase. DHT and testosterone are high-affinity AR ligands. Cytosolic AR, when bound to androgens at the ligand-binding domain (LBD) of the receptor, dissociates from multiple AR-interacting proteins, including heat shock proteins hsp90 and hsp70, undergoes a conformational change that exposes a nuclear localization signal and facilitates transport of the androgen/AR complex to the nucleus [7,8]. Chromatin recruitment of this complex to androgen response element(s) in target genes and receptor interaction with coregulators lead to transcriptional induction or repression of AR-regulated genes (Figure 1). The ensuing impact on downstream effectors is a major driver in shaping prostate functions. Elevated androgen-inducible PSA in circulation under peripheral castration (characterized by low (<20 ng/dL) blood testosterone) is a biochemical signature of advanced PCa. Kinase-mediated receptor phosphorylation is an androgen-independent, alternative path to AR activation (Figure 1).

AR activity is upregulated in advanced PCa, principally from increased AR expression due to *AR* gene amplification. Continued AR activity also occurs due to AR mutants with ligand affinity for steroids beyond androgens; AR splice variants (AR-Vs) lacking LBD; and altered AR coregulator(s) abundance/activity. Hormone therapy, chemotherapy, and emerging genome targeted therapy are principal treatments for metastatic castration-resistant prostate cancer (mCRPC). Genome alterations impacting non-AR pathways can also lead to late-stage, treatment-resistant prostate cancer.

Recent insights into genome alterations in prostate cancer that impact signaling pathways beyond the androgen receptor axis, subtypes of early stage and advanced PCa, and the dynamics of tumor-promoting and tumor-suppressive effects of immune cells are revealing new approaches to disease intervention. A generally immunosuppressive tumor microenvironment makes most prostate cancer cases unresponsive to current immunotherapy except for genome instability in two contexts. New laboratory results are suggesting that the reach of immunotherapy for advanced PCa management may be extended by targeting selected pro-tumorigenic pathways in combination therapy. This review is a synthesis of key findings on genetic, epigenetic, and molecular changes impacting PCa aggressiveness and elaborates how the information has contributed to innovations in treatment strategies and has led to genome-targeted precision medicine and cancer immunotherapy. Finally, a future scenario built on current research is presented when routine personalized treatment decision for high-risk non-metastatic and advanced metastatic prostate cancer would be a reality.

## 2. Current Prostate Cancer Therapies

### 2.1. Low-Risk and High-Risk Non-Metastatic Prostate Cancer

The risks of localized prostate cancer for recurrence and metastasis are determined from the Gleason score, tumor stage and size, and blood PSA levels. Treatment options for localized PCa deemed low-to-moderate risk entail active surveillance, radical prostatectomy (RP) by surgery, and radiation therapy (RT) with external beam radiation or brachytherapy, while watchful waiting (i.e., observation/no therapy) is the norm for very low-risk cases [9]. Individual factors, such as patient’s age and health, along with advantages and disadvantages of each option influence a treatment course [9,10] since regardless of the option selected, the low-risk disease shows a 10-year PCa-specific survival rate of nearly 99% [11]. Active surveillance helps to avoid health complications from RP and RT and therefore is recommended for younger and healthier patients. For high-risk localized PCa, surgery or external beam radiation is administered regardless of comorbidity-influenced life expectancy. RP and RT have similar efficacy. RP, however, is preferred over RT as primary monotherapy since it reduces local recurrence rates; potentially carries less risk for secondary cancer; allows the full-scale histopathological evaluation of tumor specimens; and also, potentially helps to avoid ADT [10]. Both RP and RT increase the risk for erectile dysfunction—with risk being greater for RP, and surgery leads to a higher risk of urinary incontinence, while the fecal incontinence risk is higher with RT [9]. Surgery is not an option when radiographic evidence indicates metastasis.

For histologically progressed non-metastatic PCa that is castration sensitive, a multimodal treatment design entailing ADT with or without RP and/or RT or combined with an AR inhibitor offers the greatest potential for improved long-term outcomes for patients who may harbor occult metastatic disease [9,10]. ADT induces the apoptosis of PCa cells, which leads to tumor shrinkage and inhibition of new tumor growth. The meta-analysis of a large dataset for high-risk prostate cancer showed that including external beam RT along with brachytherapy considerably shortens optimal duration of ADT and extends the period of metastasis-free survival [12]. This is a significant finding in view of the unpleasant side effects of ADT and its adverse impact on quality of life. A phase III trial (Stampede) has shown that ADT combined with an androgen synthesis blocking drug (abiraterone acetate plus prednisolone) provides improved benefits over ADT alone against non-metastatic castration sensitive PCa [13]. Therapeutic ADT includes agonists or antagonists for luteinizing hormone-releasing hormone (LHRH). The LHRH agonists, Leuprolide, Triptorelin, Histrelin, Goserelin, and two injectable LHRH antagonists, Degarelix (Firmagon®, Ferring Pharmaceuticals, Parsippany, NJ, USA) and orally deliverable Relugolix (Orgovyx®, Myovant Sciences, Brisbane, CA, USA), are FDA approved for ADT.

The development of non-metastatic CRPC is evidenced by rapidly rising blood PSA despite ADT. Disease progression in this case can be delayed by AR axis targeted therapy (ARAT) with a second-generation AR antagonist (enzalutamide, apalutamide or darolutamide) [14,15]. In a meta-analysis, a low proportion (16.5%) of non-metastatic CRPC patients was found to have avoided relapse within five years of initial therapy [12]. Of note, a 22-gene expression signature for high-risk localized PCa has recently been identified that can predict distant metastases and tumor responsiveness to second generation hormone therapy following radiation treatment in pretreatment biopsies [16]. The signature was identified based on the transcriptome analysis of archival pretreatment prostatectomy samples for which follow-up patient data over two to three decades was available. This genetic score-based predictive tool identified a subgroup of patients who did not additionally benefit from hormone therapy compared to radiation treatment only. Studies are ongoing to further validate this gene signature as a potential biomarker panel for stratifying high-risk primary PCa patients who should not be advised for additional hormone therapy beyond RT as primary monotherapy [16].

### 2.2. Targeting Metastatic Prostate Cancer

**Figure 2:**
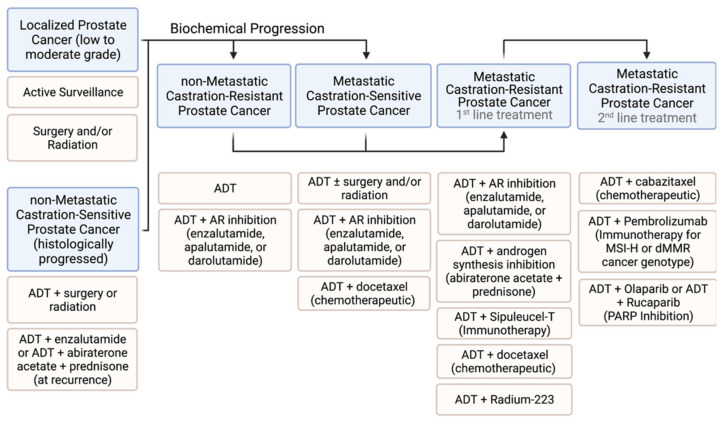
Schema for treatment modalities at progressively advancing prostate cancer stages. Boxes in blue indicate disease stages. Pink-colored boxes describe treatment choices. ADT: androgen deprivation therapy; MSI-H: microsatellite instability-high; dMMR: deficient mismatch repair.

Androgen sensitive metastatic PCa is managed by ADT monotherapy or with RT or RP. The disease invariably progresses on ADT to develop into metastatic castration resistant prostate cancer (mCRPC), when ADT together with ARAT, which employs an AR antagonist or an androgen biosynthesis inhibitor that prevents intratumoral steroidogenesis, is temporarily successful as a first line treatment. ADT plus chemotherapy (with docetaxel) is another widely used first line option. Immunotherapy using immune checkpoint inhibitors and therapy that targets the DNA repair enzyme PARP (poly (ADP-ribose) polymerase) are more recent additions to the medical armamentarium as a second line treatment against mCRPC. Common treatment practices for prostate cancer at different stages are summarized in Figure 2.

Treatment choices for high-risk, localized PCa and advanced PCa, which has metastasized, are expected to grow in the future due to the discovery of new druggable targets, many of which are under clinical evaluation. The underlying molecular and biochemical principles for each therapy shown in Figure 2 are discussed below.

#### 2.2.1. Hormone Therapy

Hormone therapy in prostate cancer implies the inhibition of androgen action in cancer cells by the systemic ablation of androgens through castration (medical or surgical), or by ARAT, which entails either inactivation of the AR due to its binding to an antagonist or suppression of intratumoral androgen biosynthesis by inactivating CYP17A1, a cytochrome P450 enzyme that catalyzes a rate limiting step in the steroidogenic pathway to testosterone and DHT production. Hormone therapy reduces cancer spread, prolongs life and controls disease-associated symptoms, such as skeletal related events. CRPC inhibition by ARAT is, however, short-lived, effective for about 4 to 5 months. Enhanced AR signaling in CRPC due to increases in AR expression and de novo androgen production leads to CRPC progression on hormone therapy. Treatment-induced stress may in part account for the upregulation of hormonal signaling in the ARAT setting. Elevated CYP17A1 in CRPC has been reported [17]. Whole genome sequencing of CRPC samples has revealed the recurrent amplification of genomic *AR* and its enhancer regions [18,19,20,21,22]. Resistance to ARAT develops also in response to functional changes in AR and is driven by mutation, coregulator alteration, and AR splice variants.

AR antagonists, upon binding to AR’s LBD, promote corepressor recruitment to DNA-bound AR, which in turn triggers regulatory events that lead to androgen action suppression and tumor growth reduction [3]. Enzalutamide (Xtandi®, Astellas, Northbrook, IL, USA), and the newer drugs apalutamide (Erleada®, Janssen, Horsham, PA, USA) and darolutamide (Nubeqa®, Bayer, Whippany, NJ, USA), are second generation non-steroidal AR antagonists approved for CRPC management. Of the three antagonists, enzalutamide is deemed inferior due to certain adverse events. Among first generation AR antagonists (bicalutamide, flutamide and nilutamide), bicalutamide (Casodex, Astra Zeneca, Cambridge, UK) is still prescribed for ADT-unresponsive PCa. TAS3681 is a novel oral AR antagonist that inhibits the full-length AR and also AR splice variants. A first-in-human study with TAS3681 on mCRPC patients unresponsive to abiraterone, enzalutamide, and chemotherapy has been conducted [23].

Abiraterone acetate (Zytiga®, Janssen Biotech, Horsham, PA, USA) is a steroidal CYP17A1 inhibitor approved for blocking the adrenal production of androgen precursors, which lowers de novo androgen synthesis in tumor tissue. Prednisone co-administered with abiraterone acetate prevents corticosteroid deficiency. ADT paired with abiraterone-prednisone is approved for metastatic castration-resistant prostate cancer (mCRPC). A phase III trial (STAMPEDE) on non-metastatic castration-sensitive prostate cancer has shown superior patient outcomes with ADT plus abiraterone acetate/prednisone compared to stand-alone ADT [13]. In a phase III trial (PEACE-1) targeting patients with de novo metastatic castration-sensitive prostate cancer, adding abiraterone/prednisone to ADT and docetaxel (a chemotherapeutic) improved radiographic progression-free survival [24].

Ongoing studies are focusing on mechanisms of ARAT resistance, predictive biomarkers for resistance, and AR pathway independent targets toward the goal of new and improved treatments for high-risk non-metastatic PCa and advanced PCa that has progressed on ARAT. AR ablation as a next-generation therapy (discussed in Section 5) holds promise in this regard.

#### 2.2.2. Chemotherapy

While not a standard treatment for early stage PCa, chemotherapy is used upon CRPC progression as combination therapy with ADT alone or ADT plus hormone therapy with second generation drugs. Docetaxel, cabazitaxel, mitoxantrone and estramustine are commonly used chemotherapy drugs. Docetaxel is a microtubules-binding taxane and a first-line chemotherapeutic that prevents nuclear translocation of AR. Castration-sensitive metastatic PCa can also benefit from ADT plus docetaxel [25]. Cabazitaxel is prescribed after disease progresses on docetaxel. Mitoxantrone and estramustine are used for palliation. Mitoxantrone is a type II topoisomerase inhibitor and estramustine is an estradiol-17β-phosphate conjugated to nor-nitrogen mustard, which is a genotoxic chemical and anti-mitotic. Estramustine disrupts the microtubular network, inhibits DNA replication and its estrogen component helps to lower the tumor issue testosterone level. A combination regimen of docetaxel plus estramustine showed a greater improvement in patient outcomes compared to docetaxel alone [26,27].

Adverse events are common with chemotherapy drugs. Docetaxel and cabazitaxel can cause severe allergic reactions and induce peripheral neuropathy, which leads to numbness, tingling or burning sensation in hands and feet. The use of mitoxantrone may lead to the development of leukemia a few years later, and estramustine increases the risk of thromboembolic events. Furthermore, since proliferation of rapidly dividing normal cells are also inhibited by chemotherapeutics similar to cancer cells, hair loss, sore throat, loss of appetite, fatigue due to low red blood count, and increased risk of infection from low count of white blood cells are commonly encountered examples of chemotherapy-induced morbidity.

Bone metastasis leading to severe morbidity, manifested as bone pain, spinal cord compression, hypercalcemia, and pathologic fractures, constitutes the most frequent bone-related complications in advanced PCa. The bisphosphonate class of drugs, such as zoledronic acid (Zometa®, Novartis, Cambridge, MA, USA) and denosumab (XGEVA®, Amgen, Thousand Oaks, CA, USA), which is a human monoclonal antibody against the RANK ligand (RANKL), are FDA approved for managing adverse skeletal events from bone metastasis [28]. Bisphosphonates prevent bone resorption by inhibiting osteoclast’s resorptive activity and osteoclast formation, and by inducing osteoclast apoptosis. Bisphosphonates also suppress the tumor growth of bone metastases. Bisphosphonate therapy prevents bone loss caused by anti-androgen therapy. Denosumab inhibits osteoclasts by inactivating the RANK-RANKL signaling pathway, which plays an essential role in the differentiation, activity, and survival of osteoclasts. RANK activation is prevented when denosumab binds to its antigen, i.e., RANKL, the ligand for RANK (receptor activator of nuclear factor kappa B) [29]. In a phase III study, denosumab was found to be superior to bisphosphonate in delaying skeletal related events for mCRPC patients; however, denosumab causes osteonecrosis in the jaws [30].

Recombinant interleukin-24 (IL24) is potentially a novel therapeutic for preventing bone metastasis since IL24 suppressed colonization of CRPC cells to bone in a mouse prostate cancer metastasis model [31]. IL24-directed metastasis suppression was further enhanced by pharmacologic inhibition of the anti-apoptotic protein MCL-1, which is a member of the BCL2 protein family. The inactivation of the AKT survival signal also heightened metastasis suppression by IL24. IL24 inhibits the RANKL/RANK signaling axis, thus blocking osteoclast differentiation, which in turn prevents cancer cell dissemination in bone marrow. AKT and MCL-1 are downstream effectors of the RANK-RANKL pathway [31]. Beyond inhibiting osteoclast formation, IL24 induces apoptosis of PCa cells. In view of its inhibitory effect on osteoclasts, recombinant IL24 is potentially useful in the management of PCa bone metastasis, especially in combination with an MCL-1 or AKT inhibitor. Small molecule inhibitors of MCL-1 and AKT are in clinical trials in combination therapy [32,33].

#### 2.2.3. PSMA-Targeted Radiation Therapy

Prostate specific membrane antigen (PSMA) is a cell surface protein expressed in PCa cells, not in normal prostate cells. PSMA is targeted for the delivery of a radioactive payload to metastasized PCa cells. ^177^Lu-PSMA-617 is one such payload where the radioactive lutetium-177 is coupled to a ligand that binds PSMA with high selectivity and delivers radiation to PSMA-expressing cells. The outcome of ^177^Lu-PSMA-617 radioligand therapy (RLT) combined with the standard of care was compared with the standard of care alone in a phase III trial when inclusion of RLT improved OS and progression-free survival (PFS) [34]. AR status may determine treatment outcome since early resistance to ^177^Lu-PSMA-617 positively correlated to *AR* amplification detected in circulating DNAs [35]. In addition, a PSMA-targeting antibody (J591) linked to actinium-225, another radioactive element, reduced PSA for 70% of mCRPC cases in a phase I trial [36]. ^225^Ac, an alpha particle emitter, is 1000-fold more potently radioactive than ^177^Lu, which emits beta particles.

In contrast to PSMA-targeting radioactive agents, which destroy PCa cells anywhere in the body, radium-223 dichloride (Xofigo®, Bayer, Whippany, NJ, USA), an injectable radionuclide, is approved for targeting bone metastasis of ADT-resistant PCa [37]. Radium-223 is absorbed readily by bone and irradiates PCa cells in the bone environment.

#### 2.2.4. Genome-Targeted Precision Therapy

The exploitation of the DNA repair gene mutation by targeting PARP (poly (ADP-ribose) polymerase) is an example of genome-targeted precision medicine. PARP inhibition therapy is approved for mCRPC with inactivating mutations of *BRCA2*, *BRCA1*, which regulate homologous recombination repair (HRR) of DNA double strand breaks (DSBs). Cases of mCRPC with alterations in other HRR pathway genes, such as *ATM*, *RAD51*, *MRE11*, *ATR*, *CHK2*, *MMR*, *MSH* and *CDK12*, are also known. Cancer genome exome sequencing identified somatically altered HRR genes in roughly 23% of mCRPC cases and germline events in 8% of cases [38,39]. Even primary PCa in the TCGA cohort showed HRR deficiency in 19% of cases [40].

**Figure 3:**
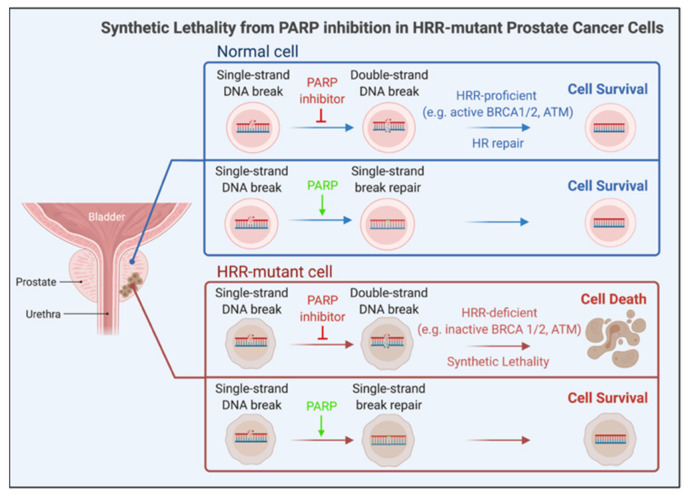
Schema for synthetic lethality from PARP inhibition in HRR mutant prostate cancer cells. Unlike HRR-proficient normal cells, HRR-mutant cells, upon PARP inactivation, accumulate DNA double-strand breaks, which cause replication block leading to cell death.

PARP inhibition is synthetically lethal for HRR-mutant cells due to the buildup of DNA DSBs leading to stalled DNA replication forks. DSBs accumulate due to unrepaired single strand breaks (SSBs). It was initially thought that, without ADP-ribosylation, XRCC1 (a DNA repair enzyme) cannot be recruited to SSBs, which then prevents activation of base excision repair (BER), causing the failed repair of DNA SSBs. A more likely model based on additional results is that the inhibitor-bound PARP is trapped onto SSB repair intermediates, especially during BER, leading to DNA DSBs. Unresolved DSBs lead to replication block and cell death. Thus, PARP inhibition induces synthetic lethality in HRR-mutant cells (Figure 3). PARP may also regulate DNA repair via an HRR-independent mechanism since hyper-activated PARP was detected in HRR-mutant cells [41].

Two oral PARP inhibitors—Olaparib and Rucaparib—which inactivate PARP1 and other PARP family members, are approved, as of May 2020, for clinical targeting of mCRPCs harboring a mutant *BRCA2* or *BRCA1* genotype [42]. The inhibitors are used in combination with chemotherapy or ARAT. PARP inhibition may also induce synthetic lethality in PCa with alterations in other HRR-relevant genes. Breast and ovarian cancers respond to PARP inhibition when harboring HRR pathway deficiency. Talazoparib, a more potent PARP inhibitor than Olaparib and Rucaparib, is under study to test for efficacy against mCRPC with aberrant HRR [43]. Talazoparib is approved for HER2-negative breast cancer with germline *BRCA1*/*BRCA2* mutation.

The functional interplay of PARP with AR has been demonstrated. For example, PARP-1 is recruited to AR-occupied genomic sites in PCa cells [44]; PARP-2, a PARP-1 paralog, enhanced AR activity by interacting with chromatin-bound FOXA1, a pioneering factor that promotes AR-mediated target transcription [45]; and a functional HRR pathway requires AR activity [46,47,48]. Diminished DNA damage response and HRR activity was detected in biopsies from patients receiving ADT plus radiation compared to radiation alone. Furthermore, ADT led to enhanced PARP activity, evidenced by increased protein parylation (i.e., poly ADP-ribosylation) in biopsies from patients on ADT [46]. Concurrent PARP and AR inhibition caused synthetic lethality in PCa experimental models [47,48]. These results suggest that ADT-associated HRR downregulation precedes the development of castration resistance, and combined PARP and AR inactivation is possibly fruitful for targeting organ-confined high-risk PCa and castration-sensitive metastatic PCa [46,47,48]. Mutant DNA repair genes identified in primary PCa support this possibility. The clinical efficacy of combined ARAT and PARP inhibition against ADT-refractive mCRPC is under investigation [47].

#### 2.2.5. Immunotherapy

Two FDA-approved immunotherapy options for mCRPC are the vaccine Sipuleucel-T (Provenge®, Dendreon, Seattle, WA, USA) and the immune checkpoint inhibitor Pembrolizumab (Keytruda®, Merck, Kenilworth, NJ, USA). Sipuleucel-T, approved in 2010 for mildly symptomatic mCRPC, is an autologous cellular immunotherapy entailing incubation of patient-derived dendritic cells, isolated from peripheral blood mononuclear cells, with the vaccine immunogen PA2024 and re-administering the immunogen/dendritic cell complex to patients [49]. PA2024 is a fusion of prostatic acid phosphatase (PAP), an immunogenic prostate-specific antigen elevated in PCa, with GM-CSF (granulocyte macrophage colony-stimulating factor). GM-CSF activates dendritic cells, which are antigen-presenting cells (APC). Activated APCs with the exposed PAP antigen stimulate cytotoxic T cells, which then recognize and kill PAP-positive PCa cells. Common adverse effects of Sipuleucel-T include fever, headache, influenza-like symptoms, elevated blood pressure, muscle ache and pain (myalgias), and abnormally excessive sweating.

Immune checkpoint inhibitors (ICIs) are approved against mCRPC and other solid tumor metastases that harbor genome alterations leading to high microsatellite instability (MSI-H) or deficient mismatch repair (dMMR) [50,51]. ICI Pembrolizumab is a human monoclonal antibody against PD-1 (Programmed Death-1), which is a cell surface protein expressed in cytotoxic CD8+T cells. PD-1 binds to tumor cell-expressed PD-L1 (Programmed Death Ligand-1), leading to T-cell suppression and immune escape of tumor cells [52]. PD-1 inhibition is regarded as more efficacious than PD-L1 inhibition since PD-L1 exists in two distinct forms [53]. Pembrolizumab is approved for mCRPC with MSI-H and/or dMMR. Pembrolizumab efficacy against mCRPCs, which show other categories of genome alterations, and which have progressed on ADT and enzalutamide, is being investigated [54]. In the KEYNOTE-365 trial, a small mCRPC cohort showed improved PSA response rate independent of HRR mutation status by the Pembrolizumab-Olaparib combination [55]. Pembrolizumab/radium-223 combination against mCRPC is under investigation (NCT03093428).

Another target for immune checkpoint suppression is CTLA-4 (cytotoxic T-lymphocyte antigen-4), which is a cell surface protein of CD8+T cells. Normally, immune blockade results from the inactivation of cytotoxic CD8+T cells following CTLA-4 binding to B7-1/B7-2 cell surface proteins that are expressed in APCs. Ipilimumab (Yervoy®, Bristol Myers Squibb, Princeton, NJ, USA) is a monoclonal antibody to CTLA-4 that disrupts the interaction between B7 and CTLA-4. T-cell receptors’ recognition of exposed antigens and major histocompatibility complex proteins on APCs and the binding of CD28 to B7-1/B7-2 lead to T-cell activation and tumor cell killing. Ipilimumab plus Nivolumab (anti-PD-1 antibody) reduced mCRPC progression in a phase II study [56].

Somatic biallelic *CDK12* inactivation, identified so far in a limited number of mCRPC cases, may also present an opportunity for immune checkpoint blockade [57]. CDK12 is a cyclin dependent kinase, which partners with cyclin K to regulate various cellular processes. CDK12-deficient tumors have elevated neoantigen burden due to focal tandem genomic duplications that generate fusion-mediated chimeric open reading frames. CDK12-/-tumors showed increased tumor infiltration of T cells, and in a small-scale clinical study, anti-PD1 monotherapy was clinically effective against these tumors [57,58].

Prostate tumor is generally adept at immune evasion except when the cancer genome alterations lead to MSI-H or dMMR, as mentioned above. For a subgroup of patients, response to immunotherapy may be augmented by reducing neoangiogenesis, a hallmark of cancer progression. For example, anti-CTLA-4, anti-PD1 and other immune checkpoint inhibition therapies lead to elevated interferon-gamma (IFNγ) in the tumor microenvironment because of increased IFNγ expression in immune cells [59,60]. IFNγ has anti-angiogenic and tumor-suppressive activity [61]. The influence of IFNγ on immunotherapy is supported by the finding that deregulated IFNγ signaling leads to deficient immunotherapy responsiveness [62]. It has been suggested based on studies in PCa cell models that high-risk PCa patients, having tumors expressing low miR-221 microRNA and elevated VEGFR2 (i.e., vascular epithelial growth factor receptor 2, an miR-221 target), may benefit from initial anti-angiogenic therapy entailing VEGFR2 inhibition with a tyrosine kinase inhibitor, e.g., sunitinib, followed by immune checkpoint inhibition [63].

A sequential treatment modality as above may suppress PCa with high VEGFR2 and low miR-221 since sunitinib-treated PC3 prostate cancer cells showed increased miR-221 expression, which is likely an escape response to VEGFR2 inhibition given that sunitinib-mediated cell proliferation blockade was prevented by ectopic overexpression of miR-221 [63]. The upregulation of miR-221 may in part contribute to the failure of clinical trials of VEGFR2 inhibition for advanced PCa [64,65]. On the other hand, sunitinib treatment led to elevated immune-related signaling in PC3 cells, including an elevated IFN-related gene signature [66]. Notably, IRF2 and SOCS3, which are repressors of IFN signaling, are miR-221 targets. Increased pro-immunogenic traits also emerged in miR-221 overexpressed PC3 cells [63]. The interrogation of prostate cancer data bases has identified a subset of high-risk primary PCa cases with tumors having abundant expression of VEGFR2, but mostly undetectable miR-221. In contrast, primary PCa samples from the TCGA cohort showed very low VEGFR2 expression [63]. Collectively, in vitro studies and clinical specimen analysis of cancer genome database have revealed a microRNA-informed epigenetic signature that can identify a subset of high-risk PCa patients who are likely to show improved response to immunotherapy with a treatment plan that includes intercepting the VEGF/VEGFR regulated angiogenesis pathway with a drug such as sunitinib.

#### 2.2.6. Summary of Approved Therapies

Current practices for managing prostate cancer are guided by the stage of malignancy, the nature of cancer genome alterations and the response of progressively advanced cancer to chemo-hormonal interventions. While low-to-moderate risk cancer is kept under active surveillance or subjected to surgery and/or radiation, a more aggressive intervention is needed for high-risk localized disease. ADT is the foundational hormone therapy and at biochemical recurrence, an AR antagonist or abiraterone acetate (plus prednisone), which inhibits intratumoral androgen biosynthesis, is added to ADT. ADT/anti-AR combination continues to be standard of care for CRPC—both non-metastatic and metastatic. Enzalutamide, apalutamide and darolutamide are second generation AR antagonists used interchangeably and their selection is based on adverse effects profiles. First-line treatment of mCRPC entails ADT plus AR axis targeted therapy—the latter employing abiraterone acetate/prednisone or an AR antagonist. Options also include combining ADT with docetaxel, a microtubule binding chemotherapy drug, or sipuleucel-T, an immunotherapy drug, or radium-223, which selectively targets PCa that has metastasized to bone. Skeletal-related events ensuing from bone metastasis of PCa are alleviated by osteoprotection with bisphosphonate therapy or denosumab-mediated antibody therapy. Both therapies inhibit osteoclast development and activity, albeit through different mechanisms. More recently, a genome-targeted precision treatment employing PARP inhibitors has been approved for second-line treatment along with ADT in the case of mCRPC, which shows deregulated DNA damage response leading to the deficient homologous recombinant repair (HRR) of DNA double strand breaks. Tumors of mCRPC patients carrying BRCA1 or BRCA2 gene mutation are approved for PARP inhibition therapy. PARP inhibition in a setting of aberrant DNA damage response, and thus deficient repair of DNA double-strand breaks, causes synthetic lethality that culminates in tumor cell death. Promising clinical responses to PARP inhibition have been observed for mCRPC patients with mutation in additional genes in the HRR pathway. This raises optimism that PARP inhibition will be an approved therapy for a wide variety of genomic mutations that interfere with HRR. Finally, immunotherapy with immune check point inhibitors (ICIs) is an approved second-line treatment for mCRPC presenting with genome alterations that cause microsatellite instability (MSI) and deficient mismatch repair (dMMR) and lead to excessive neoantigen burden and strongly immunogenic tumor cells as a consequence. These genomic alterations also promote increased PD-L1 expression and increased tumor infiltration of cytotoxic T cells.

## 3. Molecular Subtypes of Early Stage and Late-Stage Prostate Cancer

**Figure 4:**
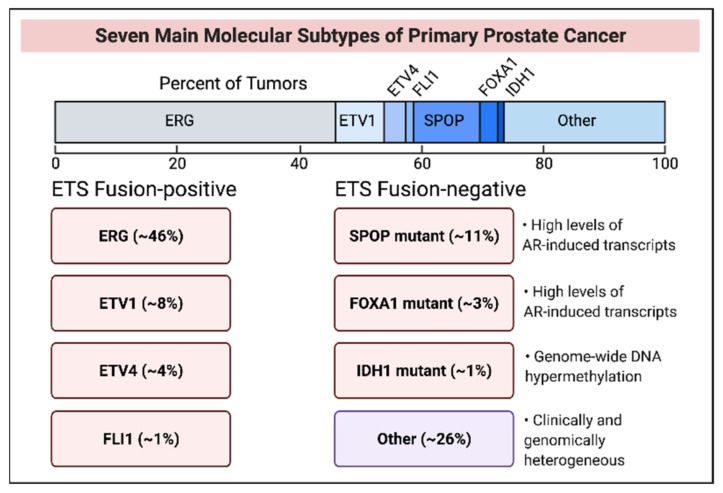
Molecular subtypes of prostate cancer. The ERG fusion subtype is most prevalent in primary PCa. The other category (~26%) associates with diverse genomic alterations, including enrichment for high copy-number alterations, DNA hypermethylation, mutations in TP53, KDM6A (encoding lysine demethylase 6A), KMT2D (encoding lysine methyltransferase 2D), and deletions of chromosomes 6 and 16. The amplification of chromosome 8 (spanning MYC oncogene) and chromosome 11 (spanning cyclin D1 encoding CCND1) is observed in tumors categorized as “other” (schema is based on Information in ref. [40]).

Genome-wide sequencing revealed distinct PCa subtypes and mechanistic studies uncovered affected signaling pathways. The TCGA cohort of 333 primary PCa cases identified 7 major molecular subtypes for 74% of early stage PCa and 26% of cases showed heterogeneous genome alterations [40]. The subtypes fall into two mutually exclusive categories—ETS fusion-positive and ETS fusion-negative (Figure 4). ETS family proteins are oncogenic transcription factors.

AR activity is variable across all cases and between seven subtypes. Sub-clonal heterogeneity within each subtype, arising from variable genome and epigenome signatures, is believed to set the disease course. Primary and metastatic PCa show a similar subtype distribution except that the IDH1-mutant subtype is absent in mCRPC. Actionable alterations affecting PI3 kinase or MAP kinase pathways were found in 20% of the TCGA cohort. The clinical translation of these findings holds promise for precision medicine.

### 3.1. ETS Fusion-Positive Subtypes

This category includes four subtypes of *ETS* gene fusion with an upstream androgen-inducible promoter. The most prevalent fusion involves the ETS protein ERG and the androgen-inducible promoter of *TMPRSS2* encoding a transmembrane serine protease. Less common promoter fusions involve other androgen-inducible genes, such as *SLC45A3*, encoding a sucrose: proton symporter [67]. Promoter fusions with the *ETS* members *ETV1*, *ETV4* and FLI1 also exist. ETS overexpression without fusion was detected in limited examples. ETS fusion-positive tumors show chromoplexy leading to extensive genome rearrangement. The deletion of the *PTEN* tumor suppressor gene, prevalent in TMPRSS2-ERG-positive PCa and a likely outcome of chromoplexy, activates the PI3K oncogenic axis. *TP53* alteration, leading to p53 tumor suppressor inactivation, is frequently detected in ERG-rearranged PCa. In *PTEN*-deleted PCa, activating the mutation of *PIK3CB* (encoding the PI3K catalytic subunit, β isoform) is a dominant driver of PI3K signaling [68,69]. AR inhibition leading to the inactivation of the androgen-inducible promoter along with PIK3CB inactivation is expected to inhibit PCa with *PTEN* deletion and activating mutation of *PIK3CB* [70]. In transurethral resections of the prostate (TURP), ETS-rearranged PCa showed increased aggressiveness [71]. The association of TMPRSS2-ERG positive cancer with a higher risk of death from PCa is inconclusive since negative and neutral associations of ETS fusions with PCa aggressiveness were also found [40].

### 3.2. ETS Fusion-Negative Subtypes

ETS fusion-negative genetic subtypes involve mutations in *SPOP* (encoding a Speckle Type BTB/POZ domain protein), *FOXA1* (encoding a pioneering factor promoting AR-mediated transactivation), and *IDH1* encoding isocitrate dehydrogenase1, which catalyzes isocitrate conversion to 2-oxoglutarate.

*SPOP* mutations are most common (Figure 4, ref [40]). SPOP regulates the proteasomal degradation of selected proteins, including the AR coactivator SRC3, which is consistent with the high AR transcriptional activity in SPOP-mutant PCa [72]. SPOP also regulates ERG degradation. SPOP interacts with the E3 ubiquitin ligase CULLIN3 and E3 ligase substrates. A role of SPOP in HRR-directed DNA DSBs repair is consistent with the association of genome instability with *SPOP* mutation. PCa cells expressing SPOP mutants are sensitized to PARP inhibitors and other DNA damaging agents [73]. *SPOP* mutations frequently associate with the somatic deletion of the genomic locus containing *CDH1*, which encodes a chromatin remodeling protein. PCa with both *SPOP* mutation and *CDH1* deletion is elevated for SPINK1, a secreted serine protease inhibitor. In the tumor microenvironment, SPINK1 promotes tumor growth and survival, and SPINK1 overexpression correlated to increased PCa aggressiveness and shortened PFS [74]. The prognostic value of *SPOP* mutation is uncertain since in one study pathological parameters did not correlate to *SPOP* mutations, while another study linked shortened PFS to reduced SPOP [40].

FOXA1 mutation has been detected in 3% of primary PCa. The molecular features of *FOXA1*-mutant and *SPOP*-mutant tumors are similar—both showing high AR activity. Primary PCa cases with *FOXA1* and *SPOP* mutations in the same clonal subpopulation are known.

*IDH1* mutation, detected in 1% of primary PCa, appears also in other cancers, and ongoing trials for *IDH1*-mutant malignancies highlight its clinical actionability. IDH1 mutation inactivates TET2, a DNA demethylating enzyme that converts methyl cytosine to hydroxymethyl cytosine leading to cytosine demethylation. IDH1 catalyzes isocitrate conversion to α-ketoglutarate, which is an essential TET2 cofactor. IDH1-mutant PCa shows robust genome-wide CpG hypermethylation. *ERG* fusion positive PCa shows a heterogeneous CpG methylation profile along with two clusters of elevated methylation. *ETV1* and *ETV4* fusions lack these clusters, showing genome-wide heterogeneous methylation. Homogeneously distributed genome-wide hypermethylation characterizes *SPOP* and *FOXA1* mutants [40].

## 4. Metastatic Castration-Resistant Prostate Cancer

The subtype distribution for mCRPC is similar to that for castration-sensitive primary, locally advanced, and metastatic PCa, except that *IDH1* mutation is absent and the mutation burden is higher. AR activity is more robust, primarily due to *AR* amplification or mutation—a feature not found prior to castration resistance. An upward of 65% mCRPC cases show actionable targets based on altered PI3K/AKT, WNT, and cell cycle and DNA repair pathways [22,75]. Other treatment-induced common genetic changes are *TP53* mutation or deletion, *PTEN* deletion, *RB1* (retinoblastoma1) loss, along with the loss-of-function mutation of *CDK12*, *BRCA2*, *BRCA1*. The PI3K/AKT pathway, which regulates tumor growth, survival, and therapy resistance [76], is activated in nearly 50% of mCRPC cases, frequently from deletion/mutation of *PTEN* [38,77]. The activation of *PIK3CA*, *PIK3CB*, *PIK3R1*, and *AKT1* is less frequent. Beyond gene deletion, PTEN loss-of-function can occur from mutation, methylation, microRNA directed post-transcriptional regulation and post-translational modification [78,79].

The reciprocal feedback inhibition of AR and PI3K/AKT signaling, demonstrated in PCa experimental models, revealed the activation of AKT upon AR inhibition and activation of AR when AKT is inactivated [80]. In our study, we showed that the dual targeting of AR and the PI3K/AKT/mTORC1 axis by the antibiotic salinomycin caused PCa inhibition in cell and xenograft models [81,82]. Salinomycin is a human stem cell inhibitor and an anti-parasite antibiotic useful in veterinary medicine. The clinical translation of combined targeting of AR and PI3K/AKT pathways yielded promising results. Ipatasertib (a pan-AKT inhibitor) plus abiraterone-prednisone improved PFS for a subgroup of mCRPC cases with PTEN-deficient, AKT hyperactivated tumors [33]. Trials with combined Capivasertib, another pan-AKT inhibitor, and enzalutamide are ongoing (NCT02525068; NCT03310541).

The aberration of DNA repair genes is found in 20–30% of mCRPC cases. Among frequently altered genes are *BRCA2*, *BRCA1*, and *ATM*, which have central roles in HRR of DNA DSBs [39]. HRR gene defects appear as somatic or germline alteration. *BRCA2*/*BRCA1* mutated mCRPCs that progress on ARAT are approved for PARP inhibition therapy. Exceptional responses to platinum-based chemotherapy for HRR-deficient mCRPC cases are known [83,84].

Alteration in mismatch repair genes—*MSH2*, *MSH6*, *PMS2* and *MLH1*—leads to deficient mismatch repair (dMMR), genome hypermutation and microsatellite instability-high (MSI-H). A total of 3–12% of mCRPC cases showed dMMR and/or MSI-H, which associate with elevated tumor-expressed neoantigens and PD-L1, and increased tumor infiltration of cytotoxic T cells [50]. MSI-H is a pan-cancer biomarker for clinical response to immune checkpoint inhibition. PD-1 inhibition by Pembrolizumab is approved for mCRPC and other solid tumors with dMMR and/or MSI-H [51].

Biallelic somatic *CDK12* inactivating mutations are more prevalent in mCRPC than primary PCa [46]. No germline mutation was found. CDK12 promotes genome stability by regulating DNA damage response genes. *CDK12*-/-tumors, which are mutually exclusive with tumors bearing ETS fusions, SPOP mutations or DNA repair deficiency, show focal tandem duplications that lead to gene fusions, chimeric coding sequences and expression of neoantigens. *CDK12*-/-mCRPC responded to anti-PD-1 monotherapy [57,58].

## 5. AR Ablation for Intercepting Advanced Prostate Cancer

Heightened AR activity and AR pathway outputs—the outcomes of increased AR expression, AR activating mutations, and AR splice variants that are activated in an androgen-independent manner—are prominent features of mCRPC. Copy number gain at the genomic *AR* locus and at a far upstream enhancer locus occurs recurrently in mCRPC and correlates to a shorter time on ARAT without any influence on OS [50]. CRPC metastases from a cohort of 197 patients showed enhanced AR signaling in 80% cases, with ~57% cases showing amplification at the *AR* locus harboring a proximal enhancer [22]. In another cohort of 101 mCRPC patients, tumors in 81% cases amplified both the enhancer-containing *AR* locus and a far-away enhancer at a 66.94 mega base upstream of the *AR* locus [21]. Copy number gain exclusively at this far upstream enhancer without *AR* locus amplification was also detected. Increased AR mRNA scores strongly associated with the two amplified loci. The far upstream amplified locus independently associated with upregulation of AR mRNAs.

An alternative approach to AR pathway inhibition is to reduce the receptor in tumor cells by its degradation via the ubiquitin-proteasomal pathway, which can be initiated by a small-molecule SARD (Specific AR Degrader) or AR-selective PROTAC (proteolysis-targeting chimera) [58,85]. One class of SARDs, which activates the proteasome machinery by having a hydrophobic degron linked to an AR ligand, caused AR ablation in experimental models, and led to sensitization of enzalutamide resistant PCa cells to AR antagonism [86]. A PROTAC is a bifunctional molecule with affinity for an E3 ubiquitin ligase and E3 substrate. By bringing the ligase near the substrate, a PROTAC facilitates substrate ubiquitination and degradation. The SARD ASC-J9^®^ degrades the wild-type AR and a clinically relevant mutant AR that confers enzalutamide resistance to PCa. ASC-J9^®^ inhibited PCa growth/proliferation and prevented AR upregulation in docetaxel-resistant CRPC [87,88]. AZD-3514 is a moderately active SARD against mCRPC, causing a decline in PSA and soft tissue tumor burden, and disease stabilization in a subset of patients [89].

Several AR PROTACs are in clinical or pre-clinical testing. The ARCC-4 PROTAC ablated cellular AR inhibited PCa cell proliferation, and degraded AR point mutants that convert enzalutamide to an AR agonist [90]. The ARD-61 PROTAC showed anti-proliferative and pro-apoptotic effects on enzalutamide-resistant CRPC in cell and xenograft models [91]. ARV-110 can induce the degradation of the wild-type AR, gene-amplified AR, and certain AR mutants in preclinical enzalutamide-sensitive and enzalutamide-resistant models. Phase I study with ARV-110 showed PSA response in two mCRPC patients who progressed on ARAT—one patient even showing reduced soft tissue tumor burden. Responding tumors harbor AR mutations that lead to *ARAT* resistance [92]. The A031 PROTAC caused AR degradation in VCaP prostate cancer cells that contain a high copy number genomic *AR*. A031 inhibited VCaP xenograft growth [93]. AR-targeting PROTACs and SARDs are promising next-generation drugs for managing AR pathway-active PCa that stops responding to current anti-AR therapy.

Unlike full-length AR, the tumor levels of the splice variant AR-V7 did not correlate to time on ARAT, although the association of AR-V7 mRNA expression in circulating tumor cells with time to ARAT resistance and clinical outcome has been reported in other studies [94,95]. Of note, a long non-coding RNA (lncRNA, designated as NXTAR) is a negative regulator of AR/AR-V7 expression while NXTAR expression itself is suppressed by androgen-activated AR [96]. A NXTAR gene-specific oligonucleotide led to the reduction in AR/AR-V7 and proliferation of PCa cells, while in a xenograft study, a small-molecule AR inhibitor restored NXTAR expression and blocked the tumor growth of enzalutamide-resistant PCa.

## 6. Lineage Switch and mCRPC Progression

*RB1* loss has emerged as a strong predictor of poor survival based on a study with taxane-naïve mCRPC patients receiving ARAT, whereas *Rb1*, *AR* and *TP53* alterations associated with a shorter time on ARAT [50]. The *RB1*-encoded tumor suppressor regulates the E2F transcription factor activity and G1→S checkpoint. A subset of ARAT-unresponsive mCRPC tumors bypasses AR pathway dependence due to lineage switch. This switch leads to a phenotype shift from luminal epithelial-like prostate adenocarcinoma to AR-independent, basal-like cells expressing neuroendocrine markers, such as synaptophysin and chromogranin A. *RB1* inactivation is the major driver of this lineage crossover to neuroendocrine-type prostate cancer (NEPC). SOX-2, a stem cell factor and regulator of neuronal progenitor differentiation, is a downstream effector of *RB1*-dependent lineage transition [97].

NEPC is a poorly differentiated mCRPC variant associated with low serum PSA and poor prognosis. A subset of NEPC histologically resembles small cell carcinoma. Variants with NEPC/small cell traits are diagnosed in up to 20% of mCRPC patients who progress on ARAT [98,99]. Prominent molecular features of NEPC/small cell carcinoma include loss of *RB1* and *TP53*, low or absent AR and AR pathway outputs, and induction of SOX2 and the epigenetic enzymes EZH2 and DNMT1. EZH2 is a lysine methyltransferase that confers a repressive epigenetic mark on histone H3 by trimethylating lysine-27 (H3K27-Me3) and DNMT1 mediates cytosine methylation at genomic context-specific CpG motifs. EZH2 is a component of polycomb repressive complex2 (PRC2). EZH2 can also coactivate AR in a PRC2-independent manner [100], which is consistent with our finding that methylation enhances AR’s transcriptional activity [101]. EZH2 inhibition reversed the lineage switch for mCRPC variants and restored enzalutamide sensitivity in enzalutamide resistant PCa models [102,103].

Aurora kinase A (AURKA), a G2→M regulating cell cycle kinase, and delta-like protein 3 (DLL3), a cell surface notch signal inhibiting ligand, are other examples of proteins enriched in NEPC and its small-cell variant compared to adenocarcinoma [98]. N-MYC oncoprotein is elevated in NEPC—a sequela to AURKA-mediated N-MYC phosphorylation. AURKA inhibition induces synthetic lethality in CRPC variants exhibiting the combined loss of *RB1* and *TP53* [98]. EZH2, AURKA and DLL3 are promising targets for which clinically relevant inhibitors are in early phase trials against NEPC and small-cell carcinoma (NCT03480646; NCT04179864; NCT01799278; NCT04702737; NCT04471727). Of note, combined inhibition of PARP and the G1 phase cyclin dependent kinases CDK4/CDK6 induced apoptosis and suppressed neuroendocrine differentiation of PCa in preclinical models [104]. Platinum-based chemotherapy, the only treatment option for NEPC and its variants, has shown limited efficacy. Clinical development of targeted inhibition of NEPC-enriched proteins is paramount if sustained control is to be achieved against CRPC variants, such as NEPC and small-cell prostate carcinoma.

## 7. Concluding Perspectives and Future Directions

Precision medicine entails diagnosis and disease management after considering individual differences in genomics and gene interactions with environment and lifestyle. The recent finding that intestinal microbiota produces androgen precursors and promotes CRPC presents the exciting possibility for antibiotics therapy in future prostate cancer management [105]. Innovations in managing mCRPC, including small cell/neuroendocrine carcinoma variants, are facilitated by a growing list of actionable genomic changes revealed by next-generation genome sequencing. Furthermore, the identification of a 22-gene expression signature, serving as a potential blueprint to managing high-risk primary PCa [16], has led to a trial focused on transcriptome profiles of pretreatment biopsy samples as the guide to treatment decision for high-risk localized disease. The efficacy of the combination therapy entailing PARP inhibition and anti-PD1 guided immunotherapy for suppressing mCRPC with gene mutation affecting DNA damage response is an example of how the cancer genome landscape might fruitfully guide clinical decision.

Immune checkpoint inhibition in monotherapy normally fails to inhibit PCa, in part due to an immunosuppressive tumor environment—the exception being mCRPC with an MSI-H or dMMR genotype that leads to enhanced cancer cell immunogenicity from high neoantigen burden. For a subgroup of mCRPC patients, tumor-suppressive immune functions may be augmented by the prior inhibition of angiogenesis. A microRNA (miR-221) has been identified as a potential biomarker for selecting patients who would possibly benefit from the neoadjuvant use of an angiogenesis-blocking drug in a cancer immunotherapy setting [63]. The clinical testing of advanced PCa expressing low miR-221 and high VEGF receptor-2 (VEGFR2) for responses to sequential treatment at first with a VEGFR2 inactivating drug, such as a clinically active tyrosine kinase inhibitor, to reduce angiogenesis followed by immunotherapy will be an important future study [63].

The inevitable progression of prostate cancer on hormone therapy leaves other strategies and actionable targets open to experimental probing and clinical validation. AR degradation by small-molecule PROTACs and SARDs as an approach to overcome AR axis blockade is in early stage clinical investigation. A clinically viable approach may be in the horizon where AR expression would be disrupted epigenetically by a long non-coding RNA known as NXTAR. The reciprocal negative regulation of gene expression for NXTAR and AR has been demonstrated, and a xenograft study showed that a small-molecule AR inhibitor restored NXTAR expression and sensitized enzalutamide-resistant prostate tumor to growth inhibition [96].

Finally, progress in CRISPR/Cas-enzymes assisted gene editing in clinical settings has ushered optimism for a future arena of prostate cancer management utilizing this cutting-edge technology. Vast possibilities for genome-targeted interventions as well as other avenues of intervention that are coming to light from mechanistic studies are advancing clinical-translational research on prostate cancer through both personalized approach and precision targeting.
